# Application of FLP-FRT System to Construct Unmarked Deletion in *Helicobacter pylori* and Functional Study of Gene *hp0788* in Pathogenesis

**DOI:** 10.3389/fmicb.2017.02357

**Published:** 2017-11-29

**Authors:** Xiaofei Ji, Ying Wang, Jiaojiao Li, Qianyu Rong, Xingxing Chen, Ying Zhang, Xiaoning Liu, Boqing Li, Huilin Zhao

**Affiliations:** ^1^Department of Pathogenic Biology, School of Basic Medical Sciences, Binzhou Medical University, Yantai, China; ^2^Central Laboratory, Huai'an First People's Hospital, Nanjing Medical University, Huai'an, China

**Keywords:** *Helicobacter pylori*, unmarked deletion, FLP-FRT, *hp0788*, cell apoptosis

## Abstract

*Helicobacter pylori* is a Gram-negative, microaerophilic bacterium associated with human gastric diseases. Further investigations on virulence genes are still required to clarify the pathogenic mechanism of *H. pylori* and the heterogeneous problem of infection. In order to develop an efficient and accurate method to study gene functions in *H. pylori* pathogenesis, an unmarked deletion method for both a single gene and a large fragment was established based on the FLP-FRT recombination system. Using this method, the gene *hp0788*, encoding an outer membrane protein (HofF), was deleted. Deletion of *hp0788* did not affect growth or motility of *H. pylori*, but reduced the adherence of the bacteria to gastric epithelial cells. The apoptosis of GES-1 cells caused by *H. pylori* infection was also reduced by the defection of *hp0788*. These suggest that *hp0788* takes part in the bacterium-host interaction and plays an important role in *H. pylori* infection. Furthermore, a large genomic fragment deletion from *hp0541* to *hp0547* in *cag* pathogenicity island was also successfully achieved using FLP-FRT method. The innovative application of the FLP-FRT recombination system in *H. pylori* to construct unmarked deletion would provide a helpful tool for further function research of putative pathogenic genes and contribute to the understanding of *H. pylori* pathogenesis.

## Introduction

*Helicobacter pylori* is a main pathogen in our gastric mucosa causing gastrointestinal diseases. It was reported that more than half of the population has been infected by this bacterium, and about 20% of them developed severe diseases according to clinical statistics (Forman, 1998; Peek and Blaser, 2002; Kato et al., 2004; Parsonnet and Forman, 2004; Sugiyama, 2004). Extensive researches have been done to identify *H. pylori* virulence factors and characterize their roles in bacterial colonization and pathogenesis. The functions of vacuolating cytotoxin A (VacA), cytotoxin associated gene A (CagA) and many important adhesins have been studied and described in detail (Ferrero et al., 1992; Haas et al., 1993; Atherton et al., 1995; Blaser et al., 1995; Backert et al., 2000, 2011; Oleastro and Menard, 2013; de Bernard and Josenhans, 2014; Zhang et al., 2014). However, the exact pathogenic mechanism, especially the different outcomes of an infection by *H. pylori*, has not been elucidated (Shanks and El-Omar, 2009; Alzahrani et al., 2014; Floch et al., 2017). It has been suggested that comprehensive effect of infecting strain virulence, host genetics and environmental factors lead to the final outcome of an infection (Yamaoka, 2008; Posselt et al., 2013). However, the current understanding of this complicated process is limited.

The outer membrane proteins (OMPs) usually play important roles in bacterial adherence, which is the first step of the colonization. *H. pylori* has a large set of OMPs, and genes encoding these proteins occupy 4% genetic sequences of the genome (Alm et al., 2000). These numerous OMPs could be divided into five families: Hop and Hor proteins, Hof proteins, Hom proteins, iron-regulated OMPs, and efflux pump OMPs. Many of these OMPs have been deeply studied on their roles in the adhesion process (Alm et al., 2000; Oleastro and Menard, 2013).

In order to explore functional genes in *H. pylori* colonization in the gerbil stomach, Kavermann and co-workers constructed 960 mutants using a signature tagged mutagenesis (STM) approach. Finally 47 genes were identified as essential for gastric colonization through screening these STM mutants, and *hp0788* was one of these genes. The protein encoded by *hp0788* was named HofF, which is a member of the Hof family. A recent report about *Helicobacter heilmannii*, one of the most predominant *Helicobacter* species in feline stomach, showed that HofF can act as an adhesin participating in bacterial adherence. The deficiency of HofF in *H. heilmannii* resulted in lower level in binding to gastric epithelial cells and gastric colonization in the stomach (Cheng et al., 2016).

Gene knockout, an important method to identify and characterize functional genes, was achieved 20 years ago in *H. pylori*, and various virulence genes have been studied through this way (Ferrero et al., 1992; Haas et al., 1993; Kahrs et al., 1995; Bauerfeind et al., 1996; Copass et al., 1997; Yuan et al., 2003). Moreover, unmarked deletion, the more accurate operation for gene knockout, was also used successfully in *H. pylori* through a sucrose-based counterselection system by Copass et al. (1997) and a *rpsL*-mediated method by Dailidiene et al. (2006).

The FLP-FRT recombination system is a useful tool for genetic engineering. It was found in *Saccharomyces cerevisiae* and has been proved to be effective in diverse bacterial species (Cox, 1983; Hoang et al., 1998; Chiang and Mekalanos, 2000; Schweizer, 2003; Stephan et al., 2004; Tracy et al., 2008; Ishikawa and Hori, 2013; Wang et al., 2014). For gene deletion, two FRT (FLP recombinase recognition target) sites were introduced into two terminals of a target gene on the same orientation. The target DNA sequence between FRT sites was further excised under the action of the FLP recombinase. The FLP-FRT recombination system could be used to excise a single gene, a large DNA segment or multiple genes repeatedly, which is the advantage compared to other site-specific mutagenesis or deletion methods used in *H. pylori* (Leprince et al., 2012; Wang et al., 2014). To our knowledge, this genetic manipulation system has not yet been used in *H. pylori*.

In this study, the FLP-FRT recombination system was applied in *H. pylori* to generate unmarked deletions. *hp0788*, a gene encoding an outer membrane protein HofF, was deleted by this method. The function of this gene in *H. pylori* pathogenesis was further analyzed through the co-cultured system of GES-1 cells and the mutant. Furthermore, a large genomic fragment deletion in *cag* pathogenicity island (from *hp0541* to *hp0547*, about 10 kbp) was obtained using this method.

## Materials and methods

### Bacterial strains, cell line, and growth conditions

*H. pylori* 26695 (ATCC700392) was used for construction of unmarked deletion in this study. *H. pylori* strains were grown on chocolate agar plates supplemented with 10% sheep's blood, and cultivated at 37°C under microaerobic conditions (85% N_2_, 10% CO_2_, 5% O_2_). Brain Heart Infusion broth supplemented with 10% fetal bovine serum (FBS) was used as liquid medium to test the growth of *H. pylori* with shaking at 100 rpm under microaerophilic conditions after inoculation. The motility of *H. pylori* was determined on soft agar (0.4%) as described by Worku and Belogolova (Worku et al., 2004; Belogolova et al., 2013). *Escherichia coli* strains were routinely cultured at 37°C in Luria-Bertani medium. Antibiotics were used at the following concentrations when needed: 15 mg/l kanamycin (Km) or 10 mg/l chloramphenicol (Cm) for *H. pylori*; 100 mg/l ampicillin (Ap) or 30 mg/l chloramphenicol (Cm) for *E. coli*. The human gastric epithelial cell line GES-1 (ATCC, Rockville, MD) was used to study the pathogenicity of *H. pylori* mutant strains. GES-1 cells were cultured with Dulbecco's Modified Eagle Medium (DMEM) containing 10% FBS. In co-cultured system of *H. pylori* and GES-1 cells, the value of multiplicity of infection (MOI) was 200:1. Bacterial strains and plasmids used in this study are listed in Table 1, and primers are listed in Table 2.

**Table 1:** Strains and plasmids used in this study.

**Strain or plasmid**	**Description or sequence**^**a**^	**References or source**
***E. coli* STRAIN**		
DH5α	Strain used for gene cloning	Clontech
***H. pylori* STRAINS**		
*H. pylori* 26695	Wild type	ATCC
*Δ0788* strain	Unmarked deletion mutant of *hp0788*	This study
Δ*0541*-*0547* strain	Deletion mutant of large genomic fragment from *hp0541* to *0547*	This study
**PLASMIDS**		
pHimarEm1	Plasmid carrying HimarEm1, Km^r^ [Em^r^]	Braun et al., 2005
pTnMax9	Plasmid carrying mini-Tn, Ap^r^ (Cm^r^)	Kahrs et al., 1995
pTSK	Plasmid carrying two FRT sites, Ap^r^ [Em^r^]	Wang et al., 2014
pCHF	Plasmid carrying FLP recombinase, Ap^r^ [Cm^r^]	Wang et al., 2014
pSJHK	Gene-targeting template plasmid, Ap^r^ (Km^r^)	Ji et al., 2016
pTSKHP	Plasmid carrying two FRT sites, Ap^r^ (Km^r^)	This study
pCHFHP	Plasmid carrying FLP recombinase, *cat-GC*, Ap^r^ (Cm^r^)	This study
pTSKHP-*0788*	A *hp0788* recombinant vector, Ap^r^ (Km^r^)	This study
pTSKHP-*0541*	A *hp0541* recombinant vector, Ap^r^ (Km^r^)	This study
pTSKHP-*0547*	A *hp0547* recombinant vector, Ap^r^ (Km^r^)	This study
pCHHP0788	Plasmid carrying *hp0788* for complementation, *cat-GC*, Ap^r^ (Cm^r^)	This study

a *Ap, ampicillin; Km, kanamycin; Em, erythromycin; Cm, chloramphenicol. Phenotypes in parentheses are expressed in H. pylori, phenotypes in square brackets are expressed in C. hutchinsonii, phenotypes not in parentheses and square brackets are expressed in E. coli*.

**Table 2:** Primers used in this study.

**Primer**	**Description or sequence**^**a**^
BssHII-KmF	CTAGCTGCGCGCTGCCGCAAGCACTCA
BssHII-KmR	GCCTTCGCGCGCGATACCCCTCGAATTGA
0788H1F	GAACGGTGGATCCGAACAGGCGTAAAGAAATCG
0788H1R	TAAGCCAGGTACCTCATAAAGGTTTCGGTAGG
0788H2F	TAGACGGTCGACCCGCCACCGATCAAGACA
0788H2R	GGACTGGAGCTCTATTAACCAAAGCCACAAAGAC
test1	TTATGGGATCAGCGAAGAAGTG
kt2	TGCCTCGTCTTGGAGTTCATTC
kt3	GTTGGCTACCCGTGATATTGCT
test4	GCTTCTGTGGATATGACTGCTT
RT0786-1	CCCTACTAATTTAGCGATCAAG
RT0786-2	AAATGCGTAACAGATTGTCTTC
RT0787-1	TTTCACCGAATTAGAGCCAACA
RT0787-2	AAGACAGATTCAAAGGCAAGGT
RT0788-1	TCAAGGCCAATACGATAAGATG
RT0788-2	AGAGCGAAACCCTAAGCCAGTA
RT0789-1	GCGATAAACGCCCTTTCTAGCT
RT0789-2	TCGCAGAAAGCAATGAAAGCAC
RT0790-1	CAAAGACGCCAAAGAAAGATTG
RT0790-2	GAGCATGATTGTTCACCCATAT
C0788-1	AATAAGGTCTAGAGTGTCTGTATTTGACTAACA
C0788-2	GGTAGCGGTACCTTAAAGGGTTGTTATTTGAA
pHel1-1	CTTGATGAGCTCGAAGCTTGTCCGTTAG
pHel1-2	CGTCTTGGTCGACTAGAAAGGGAAATG
cm-F	TCCGATGTCGACCCGGTTTTTGTTAATCC
cm-R	CACCAGGCATGCGTAACTCCTTCTTACGCCCCGCCCTGCCACTCATC
0541H1F	TGCTGCGGATCCACTTTCAACCATGTTTCAA
0541H1R	GCACCTATGGTACCCAAGCGATTTCTAACAT
0541H2F	GATAGCGGTCGACTGCGGATTAGTAAATCCCACA
0541H2R	CAACAGGAGCTCGGACATGCAGAACGATAT
0547H1F	TCTTGGGGATCCGGAAATGTTAGATGTTGAG
0547H1R	TTATTGGGTACCTTGGAGGCGTTGGTGTAT
0547H2F	TTCTGGGTCGACGTATGATAAAATTGGCTTCA
0547H2R	ATTCTGGAGCTCGTAAAATTGCGAGGTATT
T1	ACTAAGAGCAGGCGCATAGATG
T4	AATATCTGCCTTCTCGCCTTGA

a *Restriction sites on the primers are underlined*.

### Construction of gene-targeting template plasmid pTSKHP carrying FRT sites

**Figure 1:**
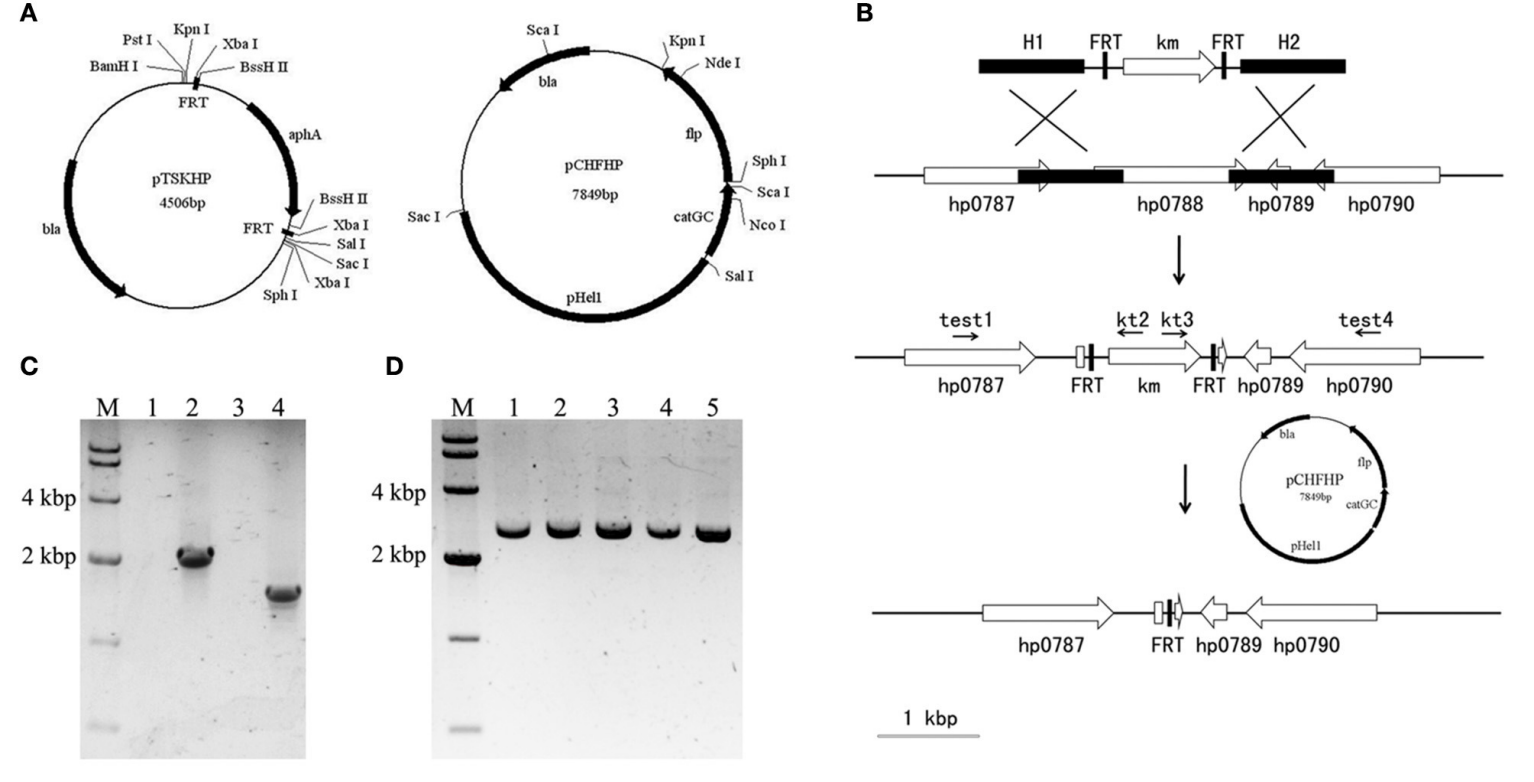
Illustration of the unmarked deletion of *hp0788* by FLP-FRT method. **(A)** Schematic representations of plasmids pTSKHP and pCHFHP. **(B)** The process of unmarked deletion of *hp0788* by FLP-FRT method. A gene-targeting cassette containing homologous arms (H1 and H2), the kanamycin-resistance gene and FRT sites was firstly transformed into *H. pylori* by electroporation. The genetic recombination at *hp0788* locus was verified by PCR with diagnostic primers (test1/kt2 and kt3/test4). Plasmid pCHFHP was then transformed into the validated transformants to delete the kanamycin resistance gene between two FRT sites. Only one FRT site was left on the locus of *hp0788* at last. Black filled boxes indicate the homologous arms; black arrows indicate the approximate locations and orientations of primers; open arrows indicate orientations and arrangements of genes; open boxes indicate residual fragment of gene *hp0788*. **(C)** Diagnostic PCR for verification of genetic recombination at *hp0788* locus. Lane M, DNA molecular weight standard (DL10000, Takara); Lane 1 and 2, PCR products amplified using primers test1 and kt2 from WT and the mutant; Lane 3 and 4, PCR products amplified using primers kt3 and test4 from WT and the mutant. **(D)** PCR verification of the deletion of *aphA* gene at *hp0788* locus. Lane M, DNA molecular weight standard (DL10000, Takara); Lane 1-5, PCR products from different tested mutants using primers test1 and test4.

Plasmid pTSK carrying erythromycin-resistant cassette flanked by two FRT sites was used as the backbone (Wang et al., 2014). As the erythromycin-resistance gene (*ermF*) on pTSK does not work effectively in *H. pylori*, a kanamycin-resistance gene (*aphA*) was used to replace *ermF* in pTSK. pTSK was digested with *Bss*H II into two fragments, and the larger backbone fragment was recovered followed by treated with alkaline phosphatase. *aphA* was amplified from pHimarEm1 with primers BssHII-KmF and BssHII-KmR, digested with *Bss*H II, and ligated with the backbone. The resulting plasmid, named pTSKHP, contains a kanamycin-resistant cassette flanked by two FRT sites with the same orientation (Figure 1A).

### Construction of the FLP recombinase expression plasmid pCHFHP

The FLP expression plasmid pCHF (Wang et al., 2014) in *Cytophaga hutchinsonii* was used as the backbone to construct the FLP recombinase expression plasmid in *H. pylori*. The pHel1 fragment (Heuermann and Haas, 1995) containing a *H. pylori*-specific replicon was amplified by PCR using the primers pHel1-1 and pHel1-2, and inserted into pCHF after digested with *Sac* I and *Sal* I, to generate pCHF-p. The chloramphenicol-resistance gene (*cat*) on pCHF could not be expressed in *H. pylori*. Therefore, another chloramphenicol-resistance gene (*catGC*) from pTnMax9 was amplified with primers cm-F and cm-R to replace *cat* after digested with *Sal* I and *Sph* I. The yielding plasmid was named pCHFHP (Figure 1A).

### Unmarked deletion of *hp0788*

The process of unmarked deletion of *hp0788* was illustrated in Figure 1B. Double-crossover recombination plasmid for *hp0788* was constructed as follows. An 828 bp fragment spanning the first 203 bp of *hp0788* and its upstream sequence was used as the upstream homologous arm (H1). It was amplified with primers 0788H1F and 0788H1R, digested with *Sac* I and *Sal* I, and then ligated into the corresponding sites of pTSKHP. Downstream homologous arm (H2) was a 1105 bp fragment containing the last 69 bp of *hp0788* and its downstream sequence. The fragment was amplified with primers 0788H2F and 0788H2R. After digested with *Kpn* I and *Bam*H I, it was also ligated into the corresponding sites of pTSKHP, yielding the plasmid pTSKHP-*0788*.

pTSKHP-*0788* was transformed into *H. pylori* by electroporation as previously described (Ji et al., 2016). The transformants were selected with 15 mg/l of kanamycin and confirmed by PCR with diagnostic primers (test1/kt2 and kt3/test4). The verified transformant cells were used as recipient cells for transforming pCHFHP by electroporation. After incubation at 37°C on chloramphenicol selective plates for 7–10 days, the transformant colonies were inoculated into fresh medium without antibiotics for the elimination of pCHFHP.

Finally, diagnostic PCR with primers test1 and test4 was preformed to verify the excision of *aphA* gene in the final transformant cells. The cells were also streaked on serum plates containing kanamycin or chloramphenicol to confirm the loss of the exogenous resistance genes.

### RT-PCR analysis

Total RNA of wild-type *H. pylori* and *hp0788* mutant was isolated using the RNApure Bacteria Kit (CWBIO, Beijing, China). Traces of DNA in extractive RNA were eliminated and first-strand cDNA was synthesized through HiFiScript cDNA Synthesis Kit (CWBIO, Beijing, China). The cDNA was used as the template to carry out PCR with primers listed in Table 2 (RT0786-1, RT0786-2, RT0787-1, RT0787-2, RT0788-1, RT0788-2, RT0789-1, RT0789-2, RT0790-1, and RT0790-2). In PCR control reactions, RNA without reverse transcription was used as the template to determine whether the RNA was free of genomic DNA.

### Complementation of *hp0788* mutant

The replicative plasmid pCHFHP was used to complement *hp0788* in the mutant. A fragment containing *Xba* I, digested from the plasmid pSJHK with *Kpn* I and *Sph* I, was inserted in the corresponding sites of pCHFHP to add the restriction enzyme site XbaI, generating pCHHPK. The fragment containing *hp0788*, 240 bp upstream of the start codon, and 28 bp downstream of the stop codon was amplified with primers C0788-1 and C0788-2. The amplicon was digested with *Xba* I and *Kpn* I and ligated into the corresponding sites of pCHHPK, yielding pCHHP0788. The plasmid pCHHP0788 was then electroporated into the *hp0788* mutant and transformants were selected by chloramphenicol resistance.

### Deletion of a large genomic fragment in *cag* pathogenicity island

The genomic fragment from *hp0541* to *hp0547* in *cag* pathogenicity island was selected for gene targeting in this study. The schematic representation of the deletion of this region was shown in **Figure 7A**. First, one of the terminal genes of this genomic fragment, *hp0541*, was deleted with the same procedure as described above. Then, the other terminal gene *hp0547*, was also subjected to unmarked deletion. Finally, the transformants were confirmed by diagnostic PCR with primers T1 and T4.

### Western blot analysis for CagA

*H. pylori* cells were harvested and washed with phosphate-buffered saline (PBS, pH 7.4). The pellets were subjected to ultrasonication. After centrifugation at 16,000 g for 5 min at 4°C, cell debris were removed and the cell lysates were analyzed by sodium dodecyl sulfate-polyacrylamide gel electrophoresis (SDS-PAGE). The proteins in the SDS-PAGE gel were transferred onto a 0.45 μm Immobilon-P PVDF membrane (Millipore, MA, USA). After blocked with skim milk, the membrane was incubated with monoclonal mouse antibody (anti-CagA) (Santa Cruz Biotechnology, CA, USA) and horseradish peroxidase (HRP)-conjugated goat anti-mouse IgG (Cowin Biotech, Beijing, China) successively. Finally, proteins on the membrane were detected by Immobilon Western Chemiluminescent HRP Substrate (Millipore, MA, USA) according to the instructions.

### Measuring the adherence of *H. pylori* to GES-1 cells

The GES-1 cells (1 × 10^5^ cells/2 ml/well) were plated in 6-well plates in DMEM medium with 10% FBS at 37°C in a 5% CO_2_ incubator overnight. Cells were washed with PBS and co-cultured with *H. pylori* (2 × 10^7^ colony forming units/ml) in 1 ml of DMEM medium for another 30 min incubation. Subsequently, cells were harvested with 1% trypsin-EDTA and collected by centrifugation. After washed three times with PBS to remove non-adherent bacteria, the remaining bound *H. pylori* on the cells were measured through plate counting and the urease test. The urease test was carried out as the procedure described by Ki et al. (2010).

### Morphology determination of GES-1 cells in the co-cultured system with *H. pylori*

GES-1 cells at a concentration of 1 × 10^5^ cells/2 ml/well were plated into 6-well plates overnight in DMEM with 10% FBS. *H. pylori* cells at mid-exponential phase were collected and added into the GES-1 cells culture at an MOI of 200:1. At the time points of 0, 8, and 16 h in co-culture, morphologic characteristics of GES-1 cells were detected through both crystal violet staining and phalloidin labeling methods. Crystal violet staining was carried out as routine procedure. In phalloidin labeling, cells were firstly fixed by paraformaldehyde for half an hour followed by washed with PBS and labeled by fluor-labeled phalloidin (Invitrogen, Waltham, MA). The images of the stained or labeled cells were finally captured through inverted fluorescence microscope (Olympus, Tokyo, Japan).

### Cell apoptosis determination

GES-1 cells were co-cultured with *H. pylori* as mentioned above. After 0, 8, or 16 h, GES-1 cells were immediately harvested to detect the cell apoptosis rate by Annexin V-FITC Apoptosis Detection Kit (KeyGEN BioTECH, Nanjing, China). According to the instructions, the assay was performed as follows. 1 × 10^5^ GES-1 cells were firstly suspended with 500 μl Annexin binding buffer containing 5 μl FITC-conjugated Annexin V antibody and 5 μl propidium iodide. Then the mixture was incubated at room temperature for 15 min in the dark. Finally, the relative number of apoptotic cells in the mixture was determined using flow cytometry (Zhang et al., 2016).

### Cell viability determination

Cell Counting Kit-8 (CCK-8) (KeyGEN BioTECH, Nanjing, China) was used to determine the cell viability according to the instructions. GES-1 cells were plated in 96-well plates with an initial density of 10^3^ cells/well, and *H. pylori* cells were added at an MOI of 200:1. After co-incubation at 37°C for 0, 8, or 16 h, 10 μl of kit reagent was added for another 3 h incubation. The absorbance at 450 nm of the sample was measured by a microplate reader.

### IL-8 secretion determination

GES-1 cells were infected with *H. pylori* strains as previously described. The supernatant of the culture was collected at 8 h, and IL-8 was measured by ELISA using an HS Human IL-8 kit (Neobioscience, Shenzhen, China), according to the manufacturer's instructions.

### Statistical analysis

Statistical analysis was carried out using *t*-test, and a *P*-value below 0.05 was considered as significant difference.

## Results

### Application of FLP-FRT system on unmarked deletion of *hp0788* in *H. pylori*

In order to apply the FLP-FRT recombination system to achieve genetic manipulation in *H. pylori*, plasmids pTSKHP carrying FRT sites and pCHFHP expressing FLP recombinase were constructed. The essential gene involved in *H. pylori* colonization, *hp0788* (Kavermann et al., 2003), was selected as a target to construct an unmarked deletion.

pTSKHP contains a kanamycin-resistance gene (*aphA*) flanked by two FRT sites. For gene targeting, homologous arms were inserted into the multiple cloning sites upstream and downstream of the resistance gene (*aphA*) on pTSKHP. After gene replacement by recombination, *aphA* flanked by two FRT sites would be inserted into the targeted site on the genome. Plasmid pCHFHP contains FLP recombinase coding gene (*flp*), which locates downstream of the chloramphenicol-resistance gene (*catGC*) directly and will be co-transcribed with *catGC*. The schematic diagrams of pTSKHP and pCHFHP are shown in Figure 1A.

As shown in Figure 1B, the *hp0788* recombinant vector (pTSKHP-*0788*) was constructed according to the procedure and transformed into *H. pylori* cells by electroporation. Kanamycin-resistant colonies were selected and verified by PCR. As shown in Figure 1C, two expected bands spanning the neighboring genes and the kanamycin resistance gene were amplified in the mutant from both upstream and downstream, which were absent from the wild-type strain. This result confirmed that *hp0788* had been replaced by the kanamycin resistance gene. Then, the kanamycin resistance gene was ejected with the transformation of the *flp* carrying plasmid pCHFHP. In order to verify the gene deletion between two FRT sites, diagnostic PCR with primers test1/test4 was performed and the results are shown in Figure 1D. A band with an expected size was amplified from each tested transformant, which indicated that the kanamycin resistance gene was lost. Sequencing analysis of the mutants confirmed the deletion of *hp0788*.

### RT-PCR analysis of the deletion of *hp0788*

**Figure 2:**
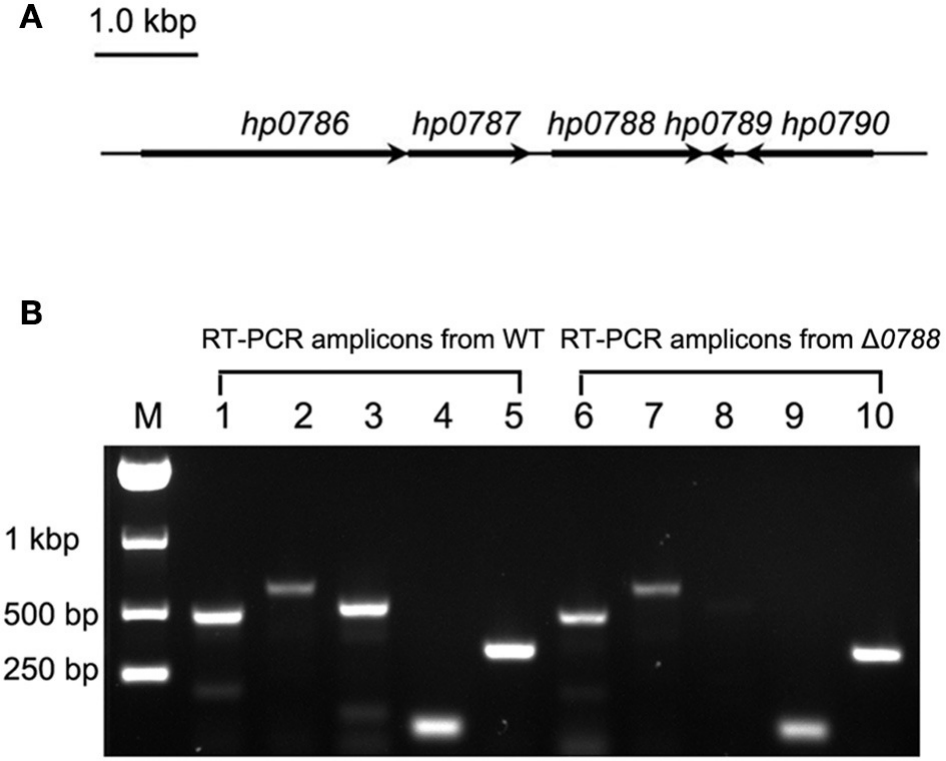
RT-PCR analysis of the transcription of *hp0788* and the surrounding genes. **(A)** Illustration of *hp0788* and the surrounding genes. **(B)** RT-PCR analysis of the transcription of *hp0788* and the surrounding genes in the wild type *H. pylori* (WT) and *hp0788* deleted mutant (Δ*0788*). PCR reactions were performed using primers RT0786-1, RT0786-2, RT0787-1, RT0787-2, RT0788-1, RT0788-2, RT0789-1, RT0789-2, RT0790-1, and RT0790-2. Lane M, Marker; Lane 1 to 5, PCR reactions performed with WT cDNA; Lane 6 to 10, PCR reactions performed with Δ*0788* cDNA. The following primers were used: RT0786-1 and RT0786-2 for lane 1 and 6; RT0787-1 and RT0787-2 for lane 2 and 7; RT0788-1 and RT0788-2 for lane 3 and 8; RT0789-1 and RT0789-2 for lane 4 and 9; RT0790-1 and RT0790-2 for lane 5 and 10.

The arrangement of *hp0788* and surrounding genes in the genome of *H. pylori* is shown in Figure 2A. To investigate whether the transcription of the surrounding genes is affected by the deletion of *hp0788*, RT-PCR was performed as previously described. In the wild type of *H. pylori*, amplicons corresponding to *hp0786, hp0787, hp0788, hp0789*, and *hp0790* were all present, while the *hp0788* amplicon was not obtained in the *hp0788* mutant (Figure 2B). This result indicated that *hp0788* was deleted. The existence of amplicons corresponding to *hp0786, hp0787, hp0789*, and *hp0790* in the mutant suggests that the deletion of *hp0788* do not affect the transcription of surrounding genes. All the amplicons were confirmed by sequencing analysis.

### The deletion of *hp0788* resulted in no significant change in bacterial growth and motility, but reduced the adherence of *H. pylori* to epithelial cells

Kavermann et al. reported that *hp0788* is an essential gene for *H. pylori* colonization in the gerbil stomach (Kavermann et al., 2003). However, the exact pathogenic role of this gene is not clear. Therefore, the characteristics of the *hp0788*-deleted mutant were identified in this paper.

**Figure 3:**
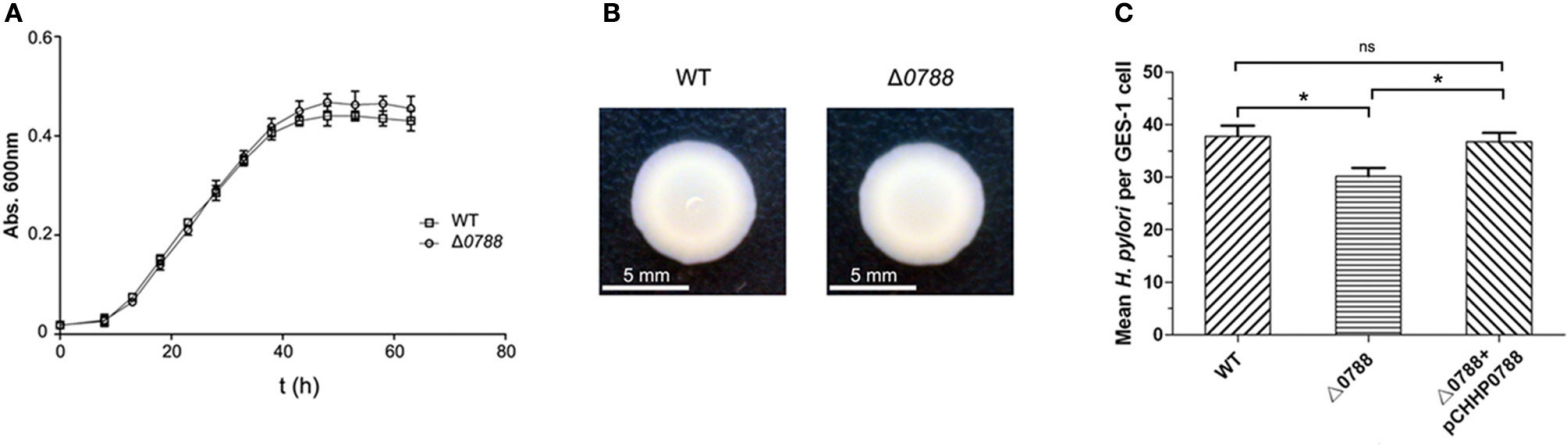
Phenotypic characteristics of the wild-type (WT) *H. pylori*, the mutant (Δ*0788*), and the complemented strain (Δ*0788*+ pCHHP0788). **(A)** Growth curves of *H. pylori* in Brain Heart Infusion broth supplemented with 10% fetal bovine serum. **(B)** The motility of *H. pylori* cells on soft agar. **(C)** The adherence of *H. pylori* to GES-1 cells. Statistically significant ^*^p < 0.05; ns, not significant.

First, bacterial morphology, growth curve, cell motility and adherence to GES-1 cells were determined to analyze the phenotypes of the *hp0788*-deleted mutant. The *hp0788* deficient strain exhibited similar morphology and structure to the wild-type strain according to the observation under transmission electron microscopy (Figure S1). As shown in Figure 3A, the mutant had a similar growth rate with the wild type except that the final cell density of the mutant was a little higher. The results of the motility assay reveal that the deletion of *hp0788* do not change the motility of the bacteria either (Figure 3B). However, the adherence of *H. pylori* to GES-1 cells was changed due to the deletion of *hp0788* according to plate counting and the urease test. For the wild type, the mean number of bacteria per cell is 37.7, while the numerical value is 30.2 for the mutant (Figure 3C). The results of urease test also illustrated the reduction in adherence of *H. pylori* to GES-1 cells (Figure S2).

To verify that the reduction in adherence was caused by the deletion of *hp0788*, complementation of the mutant was carried out as described in section Materials and Methods. As shown in Figure 3C, the reduction in adherence of *H. pylori* to GES-1 cells was almost restored in complemented strain. These results imply that HofF acts as an adhesin in the infection process of *H. pylori*, which is consistent with the previous reports (Kavermann et al., 2003; Cheng et al., 2016).

### Morphological changes of *H. pylori*-infected GES-1 cells

**Figure 4:**
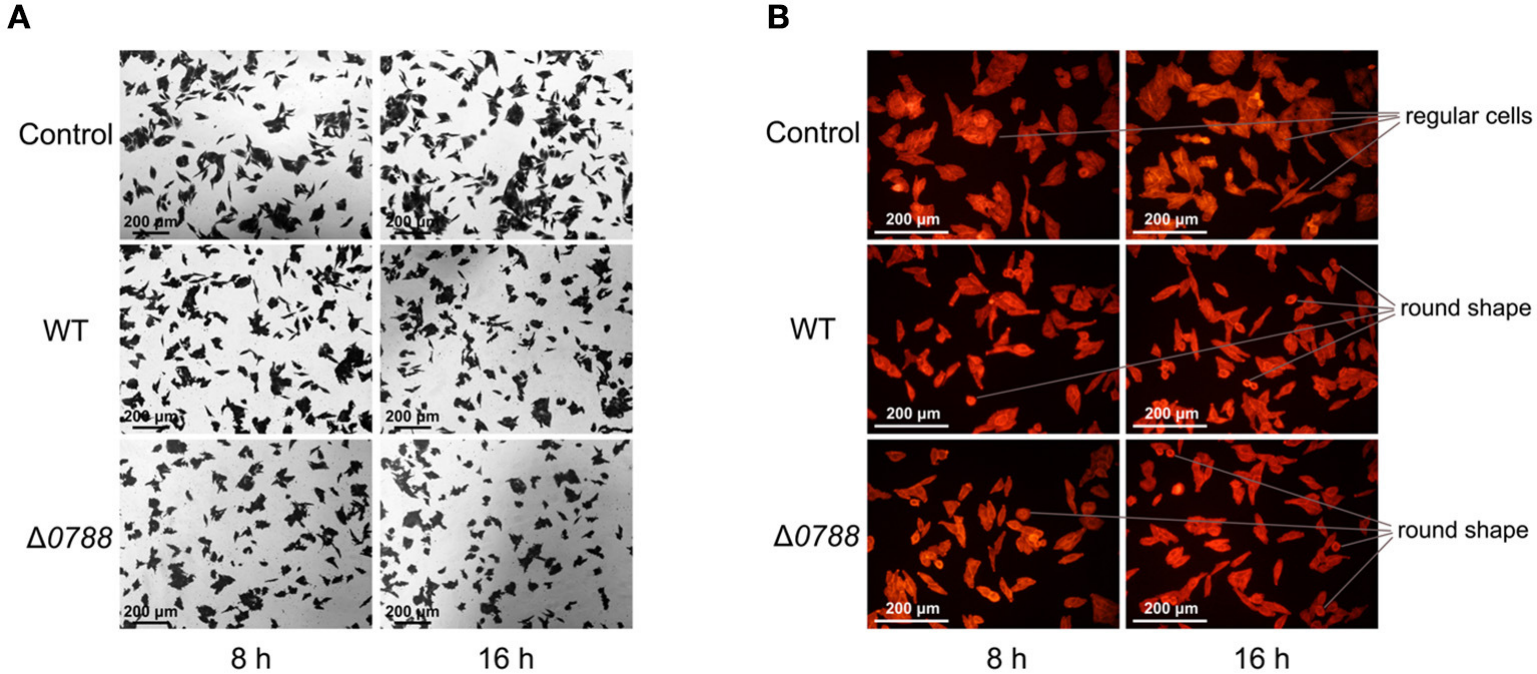
Microscopic observation of GES-1 cells in the co-incubation with *H. pylori*. **(A)** GES-1 cells were stained with crystal violet (magnification, 100×). **(B)** GES-1 cells were labeled with phalloidin (magnification, 100×). Control, non-infected GES-1 cells; WT, GES-1 cells were infected with the wild type of *H. pylori*; Δ*0788*, GES-1 cells were infected with *hp0788* deleted mutant. Regular cells and round shape cells were pointed out.

GES-1 cells were co-cultured with *H. pylori* as previously described, and morphological changes of GES-1 cells were detected. Figure 4A shows the images of GES-1 cells stained with crystal violet (magnification, 100×). Without infected by *H. pylori*, GES-1 cells exhibited agglomerated growth at 8 and 16 h. The number of GES-1 cells at 16 h was higher than the number of cells at 8 h, revealing the proliferation of GES-1 cells. After co-cultured with *H. pylori* (both the wild type and the mutant), GES-1 cells was more dispersed. The numbers of GES-1 cells at 8 and 16 h were lower than the control, which indicated that the proliferation of GES-1 cells was inhibited. Figure 4B shows the images of GES-1 cells labeled by fluor-labeled phalloidin (magnification, 200×). Without infected by *H. pylori*, GES-1 cells were regular and intact. After co-cultured with *H. pylori* (both the wild type and the mutant), some cells turned irregular and even became round. No obvious differences were observed between cells cultured with the wild type and with the mutant.

### The deletion of *hp0788* decreased the virulence of *H. pylori* to cause cell apoptosis and viability decline

**Figure 5:**
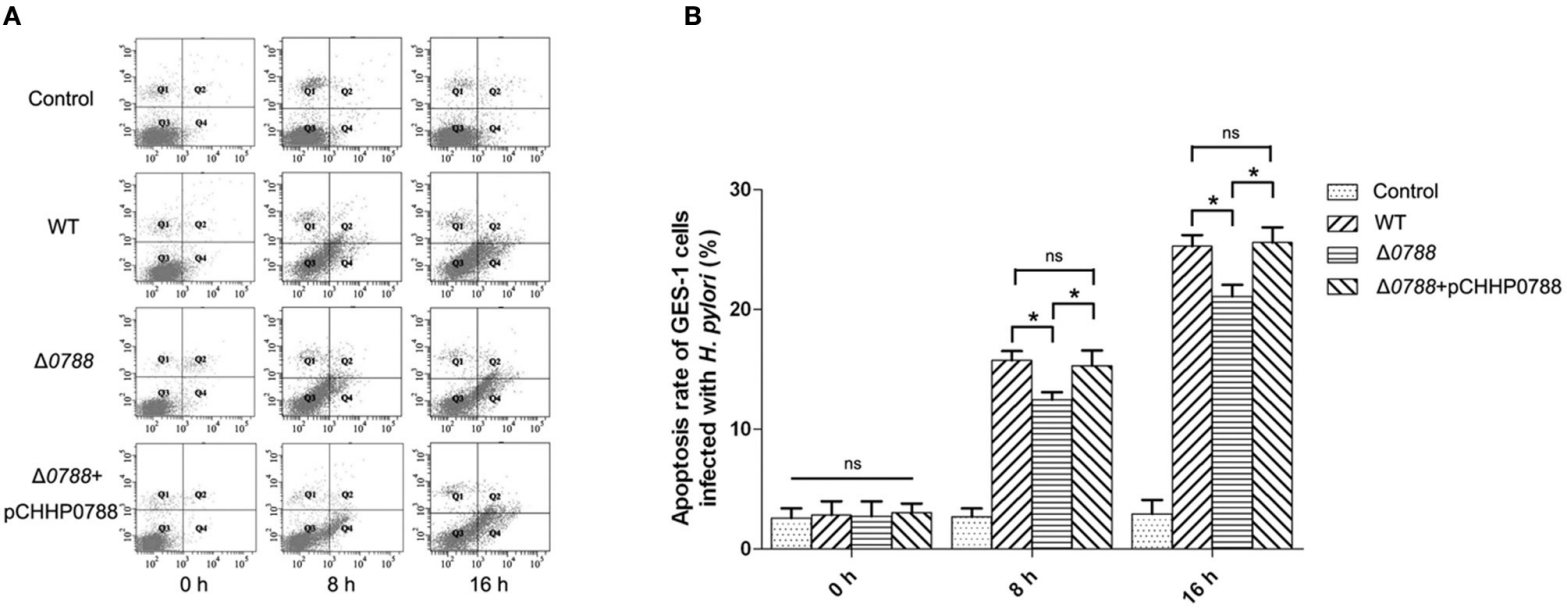
Apoptosis analysis of GES-1 cells infected with *H. pylori*. GES-1 cells were infected with the wild type (WT), Δ*0788* or the complemented strain (Δ*0788*+ pCHHP0788) at an MOI of 200:1. The apoptosis rates of GES-1 cells at different time (0, 8, and 16 h) were detected using AnnexinV-FITC/PI double staining combined with flow cytometry. Control, non-infected GES-1 cells; WT, GES-1 cells infected with the wild type of *H. pylori*; Δ*0788*, GES-1 cells infected with *hp0788* deleted mutant; Δ*0788*+ pCHHP0788, GES-1 cells infected with the complemented strain. **(A)** Representative flowcharts. Apoptotic cells occurred in the second and fourth quadrants. **(B)** Cell apoptosis rate of GES-1 cells. Apoptosis rates of GES-1 cells were calculated from the statistical analysis of apoptotic cells. Statistically significant ^*^p < 0.05; ns, not significant.

Cell apoptosis assay was performed as described in section Materials and Methods, and the relative number of apoptotic cells was determined using flow cytometry. Figure 5A shows the representative flowcharts, in which apoptosis cells occurred in the second and fourth quadrants. Apoptosis rates of GES-1 cells are calculated from the statistical analysis of apoptotic cells, which is shown in Figure 5B. Cell apoptosis rate of GES-1 cells was very low (about 4.00 ± 3.15%) without infection with *H. pylori*. After infected by the wild-type *H. pylori*, the apoptosis rate of GES-1 cells increased to 15.73 ± 7.84% after 8 h and reached 25.26 ± 5.81% after 16 h. For the co-incubation with the mutant, cell apoptosis rate increased to12.46 ± 12.3% and 21.13 ± 10.09% at 8 and 16 h, respectively. Compared with cells infected with the wild type strain, those cells infected with mutant strain showed a reduction in apoptosis. When the complemented strain was used to infect GES-1 cells, the apoptosis rate of GES-1 cells recovered to 15.29 ± 13.1% at 8 h and 25.57 ± 9.46% at 16 h, respectively. These results suggest that the deletion of *hp0788* disrupts the action of *H. pylori* to the GES-1 cells in cell apoptosis.

**Figure 6:**
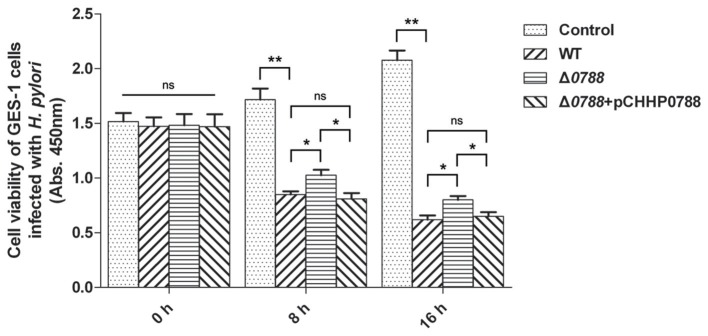
Cell viability of GES-1 cells infected with *H. pylori*. GES-1 cells were in the co-incubation with the wild type (WT), Δ*0788* or the complemented strain (Δ*0788*+ pCHHP0788). Cell viability was determined by absorbance at 450 nm after using CCK-8. Control, non-infected GES-1 cells; WT, GES-1 cells infected with the wild type of *H. pylori*; Δ*0788*, GES-1 cells infected with *hp0788* deleted mutant; Δ*0788*+ pCHHP0788, GES-1 cells infected with the complemented strain. Statistically significant ^**^p < 0.01, ^*^p < 0.05; ns, not significant.

The viabilities of GES-1 cells after infection with the wild-type *H. pylori*, the Δ*0788* mutant and the complemented strain were also comparatively analyzed. As shown in Figure 6, cell viability of the control without being co-cultured with *H. pylori* increased with time. In the samples co-cultured with either the wild-type or the mutant strain, cell viability decreased significantly. When infected by the wild type, the value of cell viability decreased to 53.43 or 41.76% relative to the initial value after 8 or 16 h. But with infection by the mutant, the value of cell viability retained 66.07 and 49.84% after 8 and 16 h, respectively. When the complemented strain was used, the value of cell viability decreased again, to 52.49 and 42.68% after 8 and 16 h, which was basically of the same level with wild type strain. These results show that deletion of *hp0788* decreases the effect of *H. pylori* on the viability of infected GES-1 cells. The tendency of cellular damage of infected GES-1 cells is consistent with the results of the cell apoptosis and viability assay.

### Deletion of a large genomic fragment in *H. pylori* based on the FLP-FRT recombination system

The *cag* pathogenicity island (*cag*PAI) is a very important region for the pathogenicity of *H. pylori* and the functions of genes within this region are still incompletely clear. So the genomic fragment from *hp0541* to *hp0547* (about 10 kbp) on *cag*PAI which contains *cagA* gene and several genes encoding the type IV secretion system was selected as the target region.

**Figure 7:**
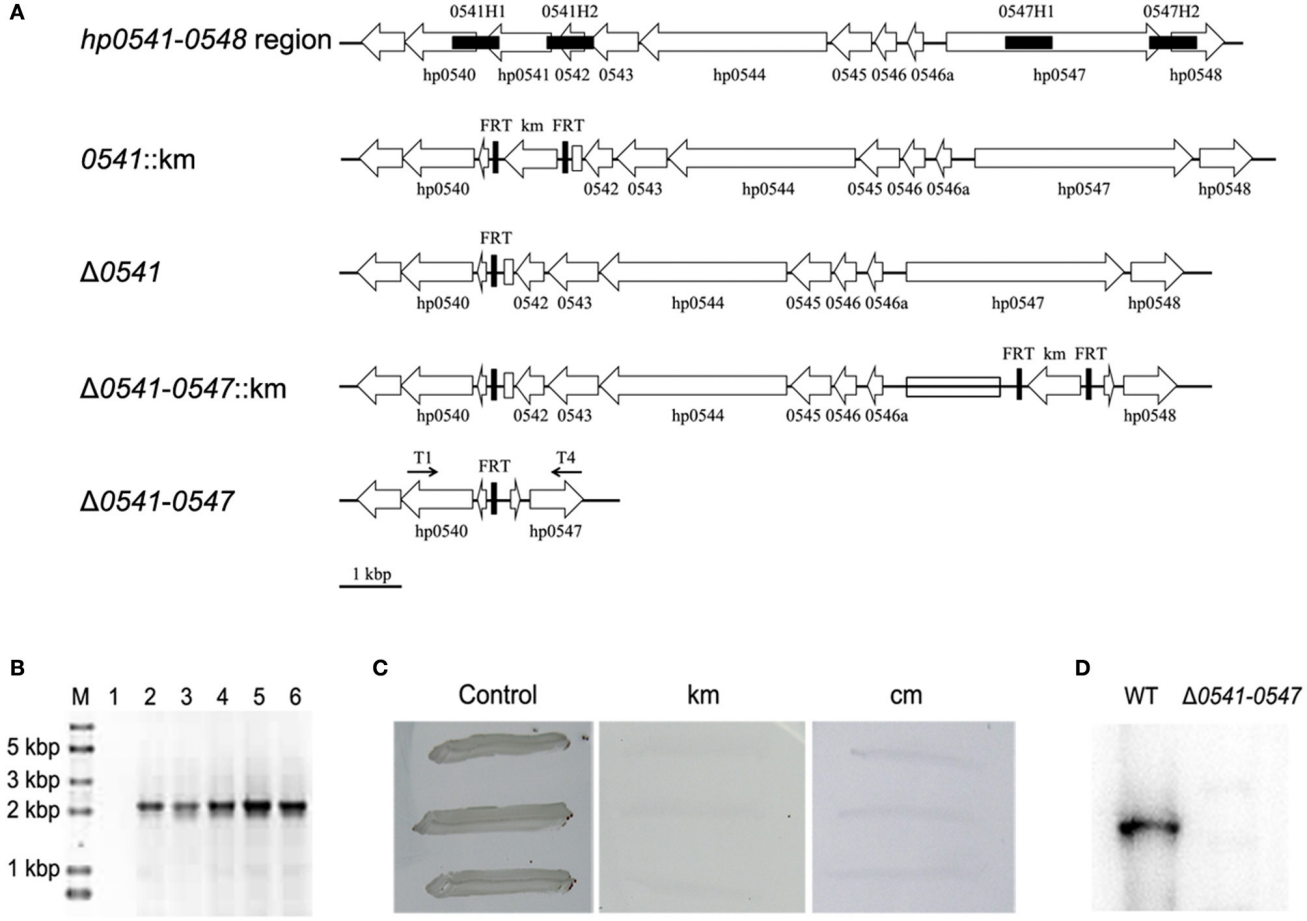
Deletion of a large genomic fragment (*hp0541-0547*) in *H. pylori* by the FLP-FRT recombination system. **(A)** Schematic representation of the deletion procedure. First, recombinant vector pTSKHP-*0541* combined with the helper plasmid pCHFHP were used to obtain the *hp0541*-deletion mutant (Δ*0541*) leaving only one FRT in *hp0541* locus. The replacement of *hp0547* with a kanamycin-resistance gene and another two FRT sites was performed with recombinant vector pTSKHP-*0547* in Δ*0541*. Finally, the helper plasmid pCHFHP was transformed into the mutant cells again to excise the genomic fragments between FRT sites, leaving only one FRT on this locus. The deletion of genetic fragments was verified by PCR with primers T1 and T4. Black filled boxes show homologous arms; black arrows indicate orientations and approximate locations of primers; open arrows show orientations and arrangements of genes; open boxes indicate residual fragments of genes *hp0541* and *hp0547*. **(B)** Diagnostic PCR for verification of the deletion from *hp0541* to *hp0547*. Lane M, DNA molecular weight standard (Trans2K Plus II DNA Marker, TransGen); Lane 1, PCR product from the wild type using primers T1 and T4; Lane 2-6, PCR products from different tested mutants using primers T1 and T4. **(C)** Antibiotic sensitivity test of the mutant cells. The mutants were streaked on fresh medium without antibiotics or containing kanamycin or chloramphenicol. **(D)** Western blot detection of CagA in the wild-type strain and the Δ*0541*-*0547* mutant.

As shown in Figure 7A, the deletion of this region was started with the disruption of *hp0541* using pTSKHP-*0541*, followed by transformation of pCHFHP to obtain the unmarked *hp0541* mutant (Δ0*541*). Then, *hp0547* was replaced by the resistant gene on pTSKHP flanked by two FRT sites in Δ*0541* mutant. The fragment containing the resistant gene and the other genes in this region will be evicted by the transformation of pCHFHP. The new junction fragments amplified from resultant colonies are shown in Figure 7B. A band with expected size was present in the mutant, while no band was amplified in the wild-type strain, mainly because the undeleted fragment is too long to be amplified using regular PCR procedure. The DNA sequencing of the amplified fragment from the mutant confirmed the deletion. After cultured without antibiotics, the mutant cells showed sensitive to both kanamycin and chloramphenicol (Figure 7C), suggesting the exogenous antibiotic-resistance genes had been eliminated.

Western blot of CagA was performed to examine the deletion on protein level. The result showed that CagA was present in the wild type, but absent from the mutant strain (Figure 7D). This result also indicated that the large fragment from *hp0541* to *hp0547* had been excised. This mutant provides a useful material for further study on the pathogenic role of the cag pathogenicity island.

**Figure 8:**
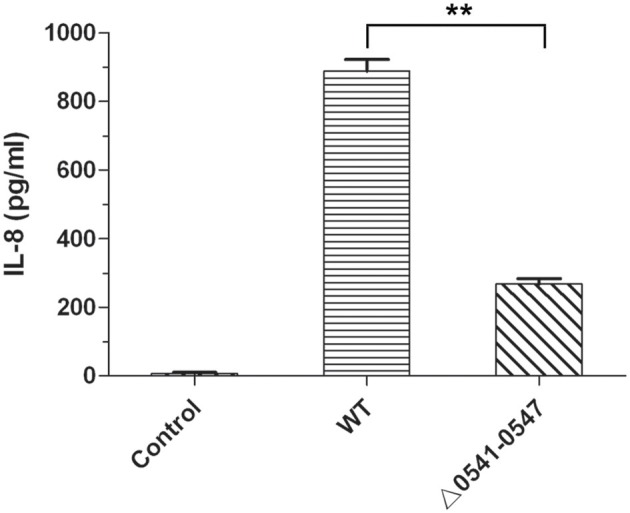
IL-8 production of GES-1 cells infected with *H. pylori*. IL-8 was measured by ELISA using an HS Human IL-8 kit. Control, non-infected GES-1 cells; WT, GES-1 cells infected with the wild type of *H. pylori*; Δ*0541*-*0547*, GES-1 cells infected with *hp0541*-*0547* deleted mutant. Data represent means and standard deviations of at least three independent experiments. Statistically significant ^**^p < 0.01.

It is clear that CagA and cagPAI could induce secretion of interleukin 8 (IL-8) in the infected cells, such as AGS cells and GES-1 cells (Fischer et al., 2001; Odenbreit et al., 2002). To further confirm the deleted mutant, IL-8 production was measured, and the results are shown in Figure 8. Consistent with the results reported by other articles (Fischer et al., 2001; Odenbreit et al., 2002; Belogolova et al., 2013), IL-8 secretion in GES-1 cells was much lower following infection with the mutant (Δ0*541*-*0547*) compared with the wild type.

## Discussion

Although, many virulence genes have been deeply studied, the pathogenic mechanism of *H. pylori* is not entirely clear (Shanks and El-Omar, 2009; Alzahrani et al., 2014; Floch et al., 2017). *H. pylori* has a high mutation rate itself and frequent genetic exchange and recombination with other *H. pylori* strains, which results in extensive genetic diversity (Suerbaum et al., 1998; Bjorkholm et al., 2001). Very little is known about the impact of genetic variation of virulence genes on disease outcomes. Therefore, more putative pathogenic genes are needed for further study to better understand the detailed pathogenic mechanism and clarify what determines different outcomes of the infection by *H. pylori*.

Construction of gene-knockout mutants is an important method for exploring novel virulence factors in pathogens. Among methods of disrupting genes, such as transposon mutagenesis (Haas et al., 1993; Kahrs et al., 1995), gene-targeting by single or double crossover homologous recombination (Ferrero et al., 1992; Bauerfeind et al., 1996; Yuan et al., 2003), unmarked deletion is the most accurate method without introduction of exogenous genes or polar effect. Copass and co-workers reported unmarked mutagenesis in *H. pylori* in 1997 through a *sacB*-mediated method (Copass et al., 1997). However, this method was proved not work well by following studies, as it is difficult to find the appropriate sucrose concentration for killing *sacB*-containing *H. pylori* cells to select expected deletion (Dailidiene et al., 2006). Dailidiene and co-workers indicated *rpsL* is a better contraselectable marker to construct unmarked deletion in *H. pylori*. With this method, the efficiency of deletion selection was considerable. However, the yield of desired deletion was obviously depending on the genome location of the targeted locus (Dailidiene et al., 2006). In addition, the eviction step in these methods was based on intrinsic homologous recombination, which usually resulted in incomplete deletion mutants. In this study, FLP-FRT recombination system was successfully applied in *H. pylori*, and unmarked deletion of a single gene was obtained through a two-step transformation. Compared with the *rpsL*-mediated unmarked deletion, the ejection of resistance genes through the FLP-FRT system is site-specific and more efficient. Moreover, the necessary step of construction streptomycin-resistant mutant in *rpsL*-mediated method is no longer needed. Unmarked deletions of several genes besides *hp0788* in *H. pylori* have also been achieved using this approach (unpublished data), which demonstrates the effectiveness of this method. Furthermore, the deletion of large DNA fragment was also achieved using this method, which has not been reported in *H. pylori*. This gene targeting method provides an efficient tool for further study of novel pathogenic genes in *H. pylori*.

The gene *hp0788* encodes a Hof protein, which belongs to an important OMP family in *H. pylori*. Kavermann and co-workers first reported that *hp0788* was essential for *H. pylori* colonization in the gerbil stomach (Kavermann et al., 2003). Recent report from Cheng and co-workers showed that the *hofF* gene is also essential for *H. heilmannii* colonization in the gastric mucosa (Cheng et al., 2016). The suspect pathway of HofF affecting *Helicobacter* colonization is that HofF can regulate MUC13 expression through IL-1β secretion, which further affects the bacterial colonization (Liu et al., 2014; Cheng et al., 2016). In this study, gene *hp0788* in *H. pylori* was deleted unmarkedly through FLP-FRT recombination system. Deletion of *hp0788* reduced the adherence of *H. pylori* to epithelial cells, which reconfirms the role of HofF as an adhesin in bacterial infection. In addition, HofF may take part in *H. pylori* pathogenesis. It was found that *H. pylori* strain lacking HofF had impaired abilities to reduce the viability and induce apoptosis in infected GES-1 cells. We speculate that HofF also acts as an effector protein playing roles in the cell-contact interaction between *H. pylori* and the host cells. To better understand the role of HofF in *H. pylori* infection, the changes in signal transmission caused by the deletion of HofF are being further studied in our lab, which may provide more insights into the mechanism of *H. pylori* pathogenesis.

## Ethics statement

This article does not contain any studies with human participants or animals performed by any of the authors. The genetic modification of *H. pylori* was used only for basic research, which was under supervision and approved by Medical Ethics Committee of Binzhou Medical University.

## Author contributions

XJ, YW, HZ, and BL: conceive and design the experiments; Perform the experiments: XJ, YW, HZ, JL, QR, and XC; XJ, YW, HZ: writing the paper; YZ, XL, and BL: Revising the paper; All authors approved the final manuscript.

### Conflict of interest statement

The method of unmarked deletion in H. pylori through FLP-FRT recombination system was submitted in patent application (CN106086054A), and the patent application is under substantive examination presently.
