# Supplementary material for: Application of FLP-FRT System to Construct Unmarked Deletion in Helicobacter pylori and Functional Study of Gene hp0788 in Pathogenesis
Source: Front Microbiol. 2017 Nov 29;8:2357. doi: 10.3389/fmicb.2017.02357 (PMC5712585; doi:10.3389/fmicb.2017.02357)
Supplement: Supplementary file 1 [file Image1.PDF]

Application of FLP-FRT System to Construct Unmarked Deletion in *Helicobacter pylori* and Functional Study of Gene *hp0788* in Pathogenesis

Xiaofei Ji[1#], Ying Wang[2#], Jiaojiao Li[1], Qianyu Rong[1], Xingxing Chen[1], Ying Zhang[1], Xiaoning Liu[2], Huilin Zhao[1]* and Boqing Li[1]*

[1]Department of pathogenic Biology, School of Basic Medical Sciences, Binzhou Medical University, Yantai, China

[2] Central Laboratory, Huai'an First People's Hospital, Nanjing Medical University, Huai'an, China

[#] these authors contributed equally to the paper

*** Correspondence:**

Boqing Li (sdliboqing@163.com) and Huilin Zhao (zhaohuilin1984@163.com)

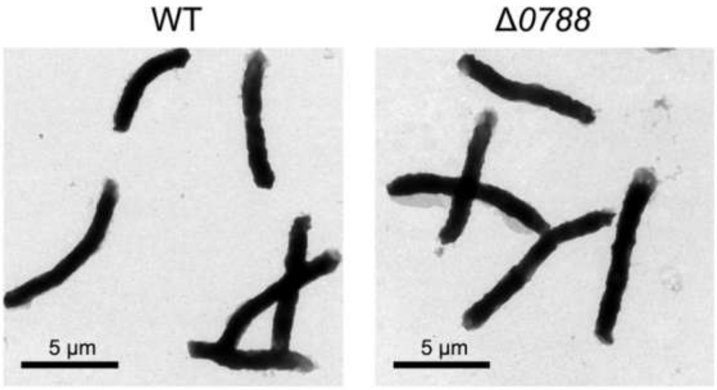

Figure S1. Transmission electron microscopy of *H. pylori* cells (magnification, 10000×). WT, the wild type of *H. pylori*; Δ*0788*, *hp0788* deleted mutant.
