# Supplementary material for: Application of FLP-FRT System to Construct Unmarked Deletion in Helicobacter pylori and Functional Study of Gene hp0788 in Pathogenesis
Source: Front Microbiol. 2017 Nov 29;8:2357. doi: 10.3389/fmicb.2017.02357 (PMC5712585; doi:10.3389/fmicb.2017.02357)
Supplement: Supplementary file 2 [file Image2.PDF]

# Application of FLP-FRT System to Construct Unmarked Deletion in *Helicobacter pylori* and Functional Study of Gene *hp0788* in Pathogenesis

**Xiaofei Ji[1#], Ying Wang[2#], Jiaojiao Li[1], Qianyu Rong[1], Xingxing Chen[1], Ying Zhang[1], Xiaoning Liu[2], Huilin Zhao[1]* and Boqing Li[1]***

[1]Department of pathogenic Biology, School of Basic Medical Sciences, Binzhou Medical University, Yantai, China

[2] Central Laboratory, Huai'an First People's Hospital, Nanjing Medical University, Huai'an, China

[#] these authors contributed equally to the paper

*** Correspondence:**

**Boqing Li (sdliboqing@163.com) and Huilin Zhao (zhaohuilin1984@163.com)**

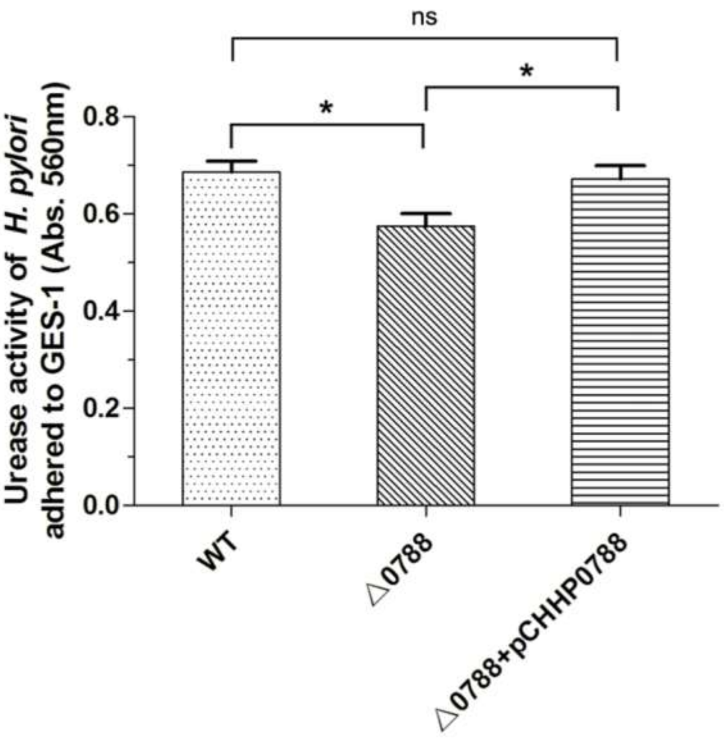


Figure S2. The adherence of *H. pylori* to GES-1 cells. Adherence of *H. pylori* to GES-1 cells was tested by urease activity. WT, the wild type of *H. pylori*; Δ*0788*, *hp0788* deleted mutant; Δ*0788*+ pCHHP0788, the complemented strain. Statistically significant $*p < 0.05$; ns, not significant.
